# Are Rank Orders Mentally Represented by Spatial Arrays?

**DOI:** 10.3389/fpsyg.2021.613186

**Published:** 2021-04-20

**Authors:** Ulrich von Hecker, Karl Christoph Klauer

**Affiliations:** ^1^School of Psychology, Cardiff University, Cardiff, United Kingdom; ^2^Albert-Ludwigs-Universität, Freiburg, Germany

**Keywords:** analog representations, spatial processing, linear orders, reasoning, mental models

## Abstract

The present contribution argues that transitive reasoning, as exemplified in paradigms of linear order construction in mental space, is associated with spatial effects. Starting from robust findings from the early 70s, research so far has widely discussed the symbolic distance effect (SDE). This effect shows that after studying pairs of relations, e.g., “A > B,” “B > C,” and “D > E,” participants are more correct, and faster in correct responding, the wider the “distance” between two elements within the chain A > B > C > D > E. The SDE has often been given spatial interpretations, but alternatively, non-spatial models of the effect are also viable on the empirical basis so far, which means the question about spatial contributions to the construction of analog representations of rank orders is still open. We suggest here that laterality effects can add the necessary additional information to support the idea of spatial processes. We introduce anchoring effects in terms of showing response advantages for congruent versus incongruent pairings of presentation location on a screen on the one hand, and the hypothetical spatial arrangement of the order in mental space, on the other hand. We report pertinent findings and discuss anchoring paradigms with respect to their internal validity as well as their being rooted in basic mechanisms of trained reading/writing direction.

## Spatial Arrays to Represent Rank Orders?

In a seminal article, Huttenlocher (1968) described what she called “spatial images” as a strategy in reasoning. Being interested in the way how people construct, and reason with, hierarchies and rank orders of various kinds, she concluded that people might “create imaginary spatial arrays when solving these problems in a manner analogous to the way they would build actual spatial arrays with real objects” (p. 553). This comprehensive claim has since been investigated by many researchers, the core question revolving around the nature of the mental representation underlying such reasoning. One possibility is to go by Huttenlocher’s conclusion and assume that spatial processes are at the basis of, or are at least necessarily associated with, reasoning about rank orders. As we shall argue in this contribution, the data so far, including self-reports and a number of behavioral paradigms, are sometimes indeed quite suggestive as indicating the use of space as a representational medium in rank-related reasoning. However, it turns out that the main signature effect in support of a spatial interpretation of the data, i.e., the so-called symbolic distance effect (SDE), can be explained in other ways, that is, without spatial assumptions. This means that the evidence for Huttenlocher’s claim remains inconclusive. In our contribution we report a series of studies in which we made an attempt at addressing this claim again from a different angle. We shall introduce a new signature effect, the Lateral Anchoring Effect (LAE) which, as we will argue, is not easily explained in other ways than spatial.

The minimal empirical situation to have in mind for rank order processing is what Huttenlocher calls the “three-term series problem” which basically exemplifies transitivity. The stimuli presented to participants are pairs of elements whereby the elements are so arranged that upon knowing two premises, a transitive conclusion may be drawn. For example, knowing that x > y, and y > z, it can be concluded that x > z. If we assume that the representation of the two premises is a spatial one, we may think of some sort of chain or sequence in mental space that is horizontally and spatially extended and looks like, e.g., x > y > z. And this is precisely what Huttenlocher’s (1968) self-report data reflect: Participants might have been given information about a number of fictitious persons, such as “Tom is taller than Bill,” or “Bill is taller than Jim.” Later on, participants would almost universally report that they constructed a spatial array of the items “in their heads,” and when asked a question about any particular pair in the order, or simply “who is the tallest,” they would then inspect the mental array to derive the correct answer. Huttenlocher’s participants also reported about the orientation of the mental arrays they created: Such arrays would either extend horizontally, with the maximum placed on the left (e.g., the “tallest”), or they would extend vertically, with the maximum placed on top. Which dimension (horizontal or vertical) was used varied to some extent, but there was a tendency to use the vertical extension for evaluative dimensions (such as “better – worse”) whereas for non-evaluative dimensions horizontal extensions were at least equally likely. Huttenlocher (1968) concluded that “subjects conceive of certain non-spatial dimensions as having particular spatial orientations, and that this spatial imagery determines how they set up ordering problems which involve these terms” (p. 552). Thus in her interpretation, mental space is used as a canvas of imagination which serves as a medium in order to reason about rank orders.

Similar experiments had been conducted by De Soto et al. (1965). Their argument in favor of spatial processing in what they called “syllogistic reasoning” was based on error rates associated with different sequences in which premises were presented. Participants had to peruse a set of two premises for 10 s, and then answer a test question. Thus, for example, they were more accurate at test after viewing “A is better than B, B is better than C,” as compared with “B is better than C, A is better than B.” The spatial explanation for this result rests again on the assumption of placing the maximum element at the origin and then constructing along an extended array in mental space. Thus, in the first pair of premises, when constructing the array, starting from an origin (A) in mental space, there is a straightforward connection to the second premise by concatenating the two premises via the common element (B). This concatenation can be easily simulated in mental space. On the other hand, in the second pair of premises, such “spatially simple” construction is not possible because the second premise has to be shifted “in front of” B, as B had been assumed to be the maximum but now is not, as revealed by the second premise. De Soto et al. (1965) also confirmed Huttenlocher’s distinction as to the use of vertical vs horizontal space, in that evaluative dimensions were reported to be predominantly associated with vertical extensions. De Soto et al. (1965) coined the term “spatial paralogic” to describe the phenomenon that by constructing mental images of integrated arrays (out of initial pairwise information), genuinely non-spatial relationships (e.g., “richer than”) were converted to relations in mental space, in order to yield new conclusions that had not been obvious in the original stimuli.

## An Analog Representation in Mental Space

The results above are just examples for the kind of data, and the kind of conclusions, that were first brought to bear on the question whether spatial mental arrays should underlie the reasoning on rank orders. As a striking characteristic of these conclusions, it is explicitly assumed that there might be something like “mental space” in which a manipulation of envisaged or imagined entities can take place, which assumption is not free of an Homunculus flavor (Kenny, 1971) when it comes to a cognitive explanation.

Another phenomenon seemed to be able to count as major empirical support for the assumption of “mental space” being involved, that is, in favor of a contribution of spatial processes in rank order processing. This phenomenon is one of the most reliable and robust effects ever obtained in experimental Psychology: the SDE. This effect means that when participants have learned a rank sequence such as *A is taller than B, B is taller than C, C is taller than D*,…etc., they will respond more accurately, and more quickly, to test queries about pairs that span wider distances on the hypothetical mental array representing the rank order, as compared to pairs of narrower distances. For example, one would respond faster and more accurately when asked who was the taller in pair AD compared to pair AC, likewise one would respond more accurately and quicker to pair AC as compared to pair AB (e.g., Potts, 1972, 1974; Smith and Foos, 1975; Trabasso et al., 1975; Moyer and Bayer, 1976; Pohl and Schumacher, 1991; Leth-Steensen and Marley, 2000). What earned the effect the qualification of “symbolic” was a finding first reported by Trabasso et al. (1975): The effect was not only observed when actual physical referents had been presented (e.g., sticks of different lengths) but also when such physical referents were represented only symbolically (e.g., by colors). This engendered the speculation that the use of space as a medium for reasoning was not limited to known psychophysical effects, as closely linked with perception (Cattell, 1902; Woodworth and Schlosberg, 1954, p.33; Welford, 1960), but was probably available to more conceptually driven processes as well, thereby encompassing dimensions that had no direct physical counterpart but were abstract in nature. The abstractness of the effect became even more clear when it was found to hold in the same way across a number of quite different, social and non-social domains, for example, when people’s representations of the letters in the alphabet were studied, or rank orders of animals of different sizes (e.g., Chiao et al., 2004), as long as the entities that were rank ordered within the domain were comparable on the same transitive dimension (Birnbaum and Jou, 1990).

Explanations for the SDE have, to varying degrees, and in combination with further assumptions, made use of the idea that when pairwise rank information is memorized, a likely representational format could be some sort of transitive array, or chain. Thus, if pairwise input information about five elements A, B, C, D, and E, for example A > B, B > C, and so on, is available, the mental representation would presumably result from a constructive process of integrating this piecemeal elements into a common array, using transitivity. The result should then be, e.g., A > B > C > D > E. Trabasso and Riley (1975) argued that by virtue of the integrative construction, pairs of elements within the array would now be associated with quantifiable distances between any pair of elements. Assuming at least an ordinal level of quantification, it is then clear that the distance between A and E should be greater than the distance between, e.g., B and C. Note that the assumption of a spatial analog representation is essential in order to arrive at this conceptualization. The SDE then explains itself by virtue of another assumption, that is, Murdock’s (1960) concept of “stimulus discriminability.” The idea here is that array elements with positions closer together on the array will be more easily confused than elements that are farther apart; in other words, the pairs differ in terms of their “relative discriminability” (Trabasso and Riley, 1975).

As Leth-Steensen and Marley (2000) have pointed out, stipulating an analog representation such as a spatial one will be particularly useful in predicting the SDE when combined with quantitative models of evidence accrual (e.g., Buckley and Gillman, 1974; Birnbaum and Jou, 1990; Link, 1990; Petrusic, 1992). In terms of implementation within the cognitive system, a basic assumption in this type of models is an iterative process of sequential accumulation of samples, not unlike the logic of the Wald test (Wald, 1947). Samples of evidence are drawn from the existing mental representation, as in case of a rank order this would be the subjectively integrated array A…E. As the quantitative values of the individual elements in the array are not error-free, the sampling process needs a minimum of instances to be drawn before a threshold value is exceeded and a response can be generated (see also Holyoak and Patterson, 1981). In predicting the SDE from such assumptions, it follows that the fewer samples are needed to reach that threshold, the greater the distance between any two elements on the analog representation is. Emprically, for example, Holyoak and Walker (1976) report that the rated distances for pairs like “decade-century,” or “second-minute” were good predictors of reaction times for correct responses, thus replicating the SDE for the natural scale of time intervals. Interestingly however, they also found that selecting the longer term of the pair *decade-century* was easier compared to the selecting the shorter term, and likewise the shorter term of the pair *second-minute* compared to the selecting the longer term. It so appears therefore, that the mental representation, apart from allowing to generate distances within pairs, also contained a surplus of semantic information about the meaning of the scale as such. This surplus information could then be seen in congruence versus incongruence with the task framing. We shall come back to this aspect later.

One fundamental question with the line of explanation just exposed is, of course, do we need to assume that the analog representation of the rank ordered information is spatial? Holyoak and Patterson (1981) in their treatment of “positional discriminability” (see also Trabasso and Riley, 1975) develop a differentiated view on this. They used a paradigm in which an array of six colored lines was shortly presented, with participants deciding which of two target lines was the leftmost or rightmost. The SDE was replicated, but Holyoak and Patterson (1981) very clearly emphasize the abstractness of any analog representation that we may have to assume as underlying the effect. First, they note that there was no evidence for any “visual-field congruity effect” (i.e., faster “leftmost” judgments for displays in the left visual field, and vice versa for “rightmost” in the right visual field), implying that the mental representation constructed did not necessarily have essential visual components. The authors go on saying that different from visual imagery, spatial extension might still be an essential feature of the analog array: “The mental representation of a linear ordering must be analogous to the representation of a spatial array. This requirement need not imply that the memory representation for an ordering is a visual image in a “pictorial” sense; however, it implies that the memorized items are represented by location distributions along a continuum” (Holyoak and Patterson, 1981, p. 1297).

Of course, with a characterization of the integrated dimensional array as abstract as this, the question arises whether space should be thought as the only possible substrate of such a dimension, and the answer is no. Given that the SDE remains to be best explained under the assumption of an analog representation of an integrated array reflecting the rank order, one may assume, for example, that the to-be compared rank positions on that analog representation are realized by differential activation levels of representational units in a connectionist model (e.g., Hintzman, 1986; Leth-Steensen and Marley, 2000). Just to sketch the way in which the representational problem is solved within the framework of the Leth-Steensen and Marley (2000) model: At the core of the model is the spread of activation (Rumelhart and McClelland, 1986) from input to output units, whereby input units correspond to presented pairs, and output units corresponding to an ordinal decision between the two elements of the pair. There is also a layer of representational units inserted in between the input and output unit layers, which fulfill the purpose of representation, that is, they associate individual elements with particular activation levels. Leth-Steensen and Marley (2000) explain that the representational units were not needed mathematically since the assumption of a linear spread of activation implies that two (or more) linear connections in sequence can be formally be replaced by a single connection (see Rumelhart and McClelland, 1986). Characteristically though, “this intermediate layer of units is conceptually essential because it is the activation on these units that provides a measure of the (transformed) internal analog representation for each discrete input stimulus” (Leth-Steensen and Marley, 2000, p. 76).

Using modeling of this type, it becomes clear that space is no longer a necessary requirement to uphold for any assumed analog representation within an explanation of the SDE. But this conclusion again entails the consequence that the SDE as a phenomenon is not indicative, or in any way diagnostic, of the existence of spatial processes that may support reasoning on rank orders, at all.

It is interesting however, that although the SDE can be modeled in ways other than spatial, there are empirical findings that still give the impression that spatial factors are most likely to have at least some explanatory value. Such evidence has been primarily accumulated in the domain of numerical representations, related to the concept of the so-called “mental number line” (Dehaene et al., 1993). One year prior to Huttenlocher’s study, Moyer and Landauer (1967) reported for the first time that in binary decisions about which of two presented numbers be the larger, response time was inversely related to the numerical distance between the two presented numbers. This numerical distance effect as since been replicated multiple times (see Sekuler and Mierkiewicz, 1977; Dehaene et al., 1990) and has led to representational assumptions in the numerical domain. The idea was that as numbers would be represented in an analog way along a cardinal dimension, those numbers at close distance with each other should be more difficult to discriminate than numbers farther apart on the dimension. Since then, the numerical distance effect has been proven one of the most robust and important effects found in the numerical cognition literature, with the effect having been explored in that literature to a much greater extent than in transitive reasoning. One particular study, as discussed below, is interesting in the present context as it again supports the idea of a spatial basis in the light of the discussion about different explanations of distance effects, either spatial or on the grounds of activation level hierarchies. The results of this study may also be of interest for our own question, related to rank orders along non-numerical dimensions:

Analyzing number comparison, Van Opstal et al. (2008) used a connectionist model as described above. They distinguished two basic ways to explain what they term the *comparison distance effect* (an effect exactly corresponding to the SDE, but in the number domain): The first they call “representational overlap,” and this is conceptually close to “positional discriminability” (Holyoak and Patterson, 1981, see above) and “relative discriminability” (Trabasso and Riley, 1975, see above): The true location of a number on an hypothesized analog array has an error distribution around it, and these distributions will create more overlap for numerically close numbers than for numbers far apart from each other. Discrimination between numbers becomes more difficult with more “representational overlap.” The second way to explain the *comparison distance effect* is called “monotonic connection” and is conceptually similar to the above-explained analog representation on the basis of different element-specific activation levels, across array elements. Critically, Van Opstal et al. (2008) point to one of their experimental results which, as they argue, can be explained by representational overlap (i.e., assuming that representation is spatial), but not monotonic connection (i.e., not in need of a spatial assumption). This particular result is related to the so-called *priming-distance effect*. The effect arises in a paradigm in which participants are asked in each trial to judge whether a presented target number is greater or smaller than a known standard number. The target number is preceded by another number that has a varied numerical distance (across trials) from the target. As a typical result, responses are facilitated when the numerical distance between both is small, as compared to large. As Van Opstal et al. (2008) argue, monotonic weight patterns cannot explain this result because the monotonic connection view does not make predictions concerning any sort of priming effect. On the other hand, as the authors submit, the effect is well explained by assuming that representational overlap causes co-activation of the target on viewing the prime, due to the assumed spatial closeness between prime and target number on the number line.

In the present context, the above result is interesting because it retains the possible hypothesis of a spatial representation underlying the reasoning on rank orders along abstract dimensions, as well. Whilst we have to acknowledge that the result occurred in the context of the mental number line and may as such not be completely generalizable to the SDE along abstract dimensions, the conceptual (and empirical) parallels appear considerable enough to pay attention to it. Indeed, in the numerical domain there is meanwhile overwhelming evidence pointing toward the idea that reasoning with numbers does rely on spatial representations (Chatterjee, 2001; Gevers et al., 2005; Zebian, 2005; for a review see Hubbard et al., 2005). However from the present perspective, the question is to what extent the same agreement can be furnished for order representations along abstract dimensions, which do not necessarily imply cardinal quantification, or any clear mapping on numbers. In this respect, it is interesting to note that the representational overlap view has been argued as being applicable to non-numerical stimuli, too, in the sense of them being represented on a spatial continuum (Jou and Aldridge, 1999).

As a summary therefore, we can say that although the SDE cannot sufficiently serve as a useful signature effect with respect to spatial processes underlying reasoning on rank orders, there is still evidence pointing to the possibility of such processes being engaged, nevertheless. The task is to find a paradigm that is, in this respect, more diagnostic than the SDE.

## Neurophysiological Explanations

The above models all have in common that they assume some kind of analog representation of rank orders, but they are inconclusive about whether the dimensional substrate of the dimension is to be assumed as spatial, as compared to, for example, a dimensional representation of neural excitation levels associated with the to-be-ranked elements (more amenable to a connectionist model). The models for behavioral data that rely on the SDE as primary evidence are therefore not a sufficient basis for gaining evidence by which the idea of spatial processes could be supported independently of self-report data. At the same time, our question is precisely about alternative behavioral data that would indeed provide such support, thereby aligning with Huttenlocher’s self-report observations. One alternative way of envisaging such empirical support comes from the consideration of neurophysiological results. Irrespective of the caveats discussed above, the SDE now comes into the play again as an indicator of spatial processing, provided one can demonstrate that the obtained activation patterns in particular targeted areas of the cortex have been previously associated, in other paradigms, with spatial processing (see Knauff, 2013, for an approach that integrates theoretical views on how visual and non-visual spatial areas in the brain might be involved in the construction of mental models and simulations). So, for brain research, it still seems promising to investigate parallels between neurophysiological and behavioral data with respect to replicating the SDE, as this might give support to the idea that spatial simulations may in some way support reasoning with mental models about non-spatial content.

In similar order-constructing paradigms as discussed so far, cortical activation in prefrontal and parietal areas has been identified; areas for which it is known that they are contributing to working memory performance, and support spatial processing. These experiments used procedures of learning and reasoning on series of transitively ordered non-spatial stimuli are similar to the ones described above (Christoff et al., 2001; Goel and Dolan, 2001; Acuna et al., 2002; Heckers et al., 2004; Van Opstal et al., 2009). For the numerical distance effect, Ansari et al. (2005) showed it was correlated to the activation of posterior parietal areas, in particular the inferior parietal lobule (IPL) and the superior parietal lobule (SPL) which both have shown increased activation with relational reasoning and the processing of spatial relations (Kroger et al., 2002), although the same areas, interestingly, are also associated with quantitative comparisons in general (Fias et al., 2003; Pinel et al., 2004). Most relevant to our discussion, Zalesak and Heckers (2009) found activation in these parietal areas during a transitive inference task in which participants were asked to indicate the relation between two terms (e.g., A > C) after they had learned a set of two premises involving a middle term (e.g., A > B and B > C). Whilst IPL activation had previously been thought to exclusively subserve number comparisons (Sandrini et al., 2004), the authors now use this finding to license a generalization from the previous numerical distance results to non-numeric, symbolic materials: “We suggest that the parietal activation reported here for the SDE is due to the greater difficulty in comparing items that are closer together on a spatial continuum that represents the sequence” (p. 29). Notably, Zalesak and Heckers (2009) also advocate a distinction between the role that parietal areas may play in terms of analog representation, and the role that other brain areas, such as the dorsolateral prefrontal cortex, may play in terms of manipulating and integrating the sequentially presented piecemeal information which is used, in the first place, to construct the representation (see also Acuna et al., 2002).

This also converges with other evidence. A particularly strong point with respect to generalizing distance effects from the numerical to the non-numerical domain was made by Zorzi et al. (2011). These authors used a more fine-grained technique of fMRI whereby the analysis (using multivariate classifiers) allowed one to distinguish between tasks referring to number comparisons versus letter comparisons. Using this method, they could identify distinct sets of neurons within the intraparietal sulcus that were activated in each case. Importantly, in both cases, distance effects were observed. This extends earlier work by Fias et al. (2007) who had shown that the anterior intraparietal sulcus was responsive to numbers as well as letters. Whereas this latter work already implied that abstract, non-numerical knowledge was being processed in these areas as well, only the use of multivariate classifiers made it possible to reveal distinct neuron populations being responsive in each case.

In one of our own studies (Hinton et al., 2010, using abstract symbols and artificial “more than” vs “less than” relations) we found activation patterns in the bilateral parietal areas that were rank ordered such that wider distanced pairs were associated with less activation. Corresponding behavioral data in that study showed test items related to wider distanced pairs were easier than items involving narrower distances (a replication of the SDE, see also Zalesak and Heckers, 2009). Most participants later indicated that in order to solve the task they had tried to form a mental chain. Some authors (e.g., Fias et al., 2003; Walsh, 2003; Barsalou, 2008, see also Dehaene et al., 1998) have argued that the parietal lobe might be functionally related to a general simulation and magnitude comparison device although others have challenged the assumption that such functionality would immediately imply a common mental representation of magnitude (Cohen Kadosh et al., 2008). Another study focusing on the parietal lobe, using a syllogistic reasoning task, found increased activity particularly in the learning phase, that is, when premises were to be integrated (Reverberi et al., 2010, but see above). This may imply spatial processes to be engaged predominantly during this integrative phase, which, on the other hand could point to the possibility, different from the view implied by the Zalesak and Heckers (2009) results, that although spatial areas might be involved in the construction of a mental model, this does not necessarily mean that spatial processing also manifests itself during reasoning later on, that is, at the behavioral level during test.

One criticism of the above chains of argumentation is the so-called reverse-inference problem (Poldrack, 2011; Hutzler, 2014) which is likely to arise in cases where a certain brain region is labeled to identify the activation or execution of one particular cognitive process. Whereas a standard way of drawing an inference from neurophysiological data may be described as “if process A is engaged, then brain area B is active,” a common reverse inference logic can be found in the literature that uses three steps, as follows: 1. In the present experiment we administered task 1, and area B was active; 2. In previous experiments, when process A was probably engaged, that same area B was active; and 3. Therefore, the result of area B being active in the present experiment indicates that task 1 actually engages process A. This reverse logic appears problematic because in terms of the relation between brain regions and brain functions, there is, in general, no one-to-one mapping: It is rare that a particular brain region should be activated exclusively by one particular cognitive process. It is for this reason that further behavioral data, independent of self-report, are useful or even necessary in order to answer our main question. Can we find evidence for the contribution of spatial processes during reasoning on rank orders?

However in neurophysiological research as well, efforts have since been made to overcome the limitations imposed by the logic of reverse inferencing, and the results are overall in favor of a positive answer to this question. Representational similarity analysis (RSA) has been developed as a method of relating the similarity structure in a given task to patterns of activation across a number of areas in the cortex (Kriegeskorte et al., 2008). In the case of linear order reasoning, Alfred et al. (2018) were able to show that for a linear order on a physical dimension (A taller than B, B taller than C, etc.), the hierarchy structure in terms of a dissimilarity matrix based on pairwise item comparisons – as representing the predicted mental model, and analogous to multidimensional scaling – was isomorphic to an activation pattern involving the intraparietal sulcus, precuneus, and inferior frontal gyrus. The spatial interpretation was confirmed using reverse-inference patterns as well. This type of analysis represents progress in that a finer-grained analysis based on specific predictions from the modeled task structure is possible. Predictions of this kind can be queried as to their physiological implementation, and as such the technique provides more direct evidence in support of spatial processes at work in ordinal reasoning than before. In a later study, the authors used the same technique, and stimuli that were not inherently spatial, including non-meaningful comparators such as “vilchiness,” in order to demonstrate the abstract nature of the mapping onto a spatially based mental model (Alfred et al., 2020). Across a diversity of stimulus types in this study, activation patterns isomorphic to the hypothesized mental model structure were found in the superior parietal lobule and the anterior frontal cortex.

Also, more recently, independent localizer tasks have been used to overcome the reverse inference problem. In this technique, an independent task is used to validate interpretations from functional MRI observations. In a study by Mathieu et al. (2015), an independent spatial maintenance task (comparison of dot distributions in a plane) was used to validate the spatial interpretation of their main task. They found convergent activation patterns in this task, and in their main task (constructing a linear order between four imaginary characters), involving several frontal and parietal regions (especially the Superior Parietal Lobule).

Based on the pattern of these recent neurophysiological findings, spatial interpretations of mental models about linear orders can be seen as receiving support. At the same time, convincing evidence on the behavioral side is accumulating as reviewed next.

## The Lateral Anchoring Effect (Lae)

The so-called SNARC effect (“spatial numerical association of response codes”) has been demonstrated for numerical stimuli, presumably giving evidence for a number line simulation in mental space (Gevers et al., 2005; Hubbard et al., 2005). The effect, first documented by Hinrichs et al. (1981) was coined that way by Dehaene et al. (1993) when conducting a series of experiments in which the parity status of a number had to be evaluated (odd vs even). The authors found that participants were quicker for small numbers when the response had to be given with the left hand (i.e., “odd” or “even,” depending on counterbalanced response mapping) than with the right hand, and vice versa were quicker to respond to large numbers when the correct response was to be made with the right hand. Notably, cardinality or magnitude of the number was not part of the logic underlying the task, so the laterality effect appeared to be independently based on an automatic activation of magnitude as mapped onto a spatial dimension, the “number line.” According to comparative studies, in Western countries (cultures with left-to-right reading and writing systems), this line is assumed to extend from left to right with increasing magnitude. However, in countries with a culture of right-to-left-reading and writing it would extend from right to left (Tversky et al., 1991; Dehaene et al., 1993; Chatterjee, 2001; Maass and Russo, 2003; Zebian, 2005). Accordingly, English speaking participants can respond faster to an “odd vs even” query if the queried number is large and the response is made with the right hand, compared the left side; the opposite hand preference holds for a small number.

The SNARC paradigm was used by Gevers et al. (2003) in order to generalize the finding to the non-numerical domain. They had their participants judge the position of the months in the year, as coming before or after July. Months from the beginning of the year were associated with faster reactions from the left as compared to the right hand, whereas this laterality effect was spatially reversed for months more close to the end of the year.

In light of our question, these results yield three important aspects. First, the interpretation of results obtained within the SNARC paradigm is usually in terms of number-space associations. Here, now, was evidence that non-numeric symbolic sequences, too, had spatial associations. Secondly, these spatial associations could easily be interpreted as *dimensional*, that is, space could be assumed to represent, in this case, an underlying dimension, in this case time (as much as previously, the ascending number line). Thirdly, the dimensional representation apparently contains more information than just defining the spatially extended dimension itself. As a surplus, the representation also implies an asymmetry, in that early months seemed to be represented left, whereas later months were “located” right. This of course dovetails with Huttenlocher’s self-reports to the extent that the majority of her participants described the order they were constructing as starting with the maximum either on top moving down, or with the maximum on the left side, moving to the right (these being Western participants). Thus, it appears that this kind of asymmetry, as confirmed using the SNARC methodology, is a systematic feature that might have to do with the semantic properties of the dimension (recall the asymmetric surplus information we discussed above in the context of the data by Holyoak and Walker, 1976).

The SNARC paradigm as discussed above, numerical and non-numerical, is defined in terms of an interaction between magnitude and side of a motor response, so basically has a sensomotoric constraint. However, additional evidence suggests that the effect is likely based on a high-level, central representation of magnitude, as it replicates across visual and auditory modalities, numerals, number words, and dice patterns (Nuerk et al., 2005). As such, the effect was perhaps not necessarily tied to particular sensomotoric conditions. The idea was therefore to see whether it was possible to elicit a similar laterality effect by juxtaposing congruence against incongruence between a dimensional representation on the stimulus side on the one hand, and an assumed mental model representing the dimension within mental space, on the other hand. Therefore, we designed a paradigm based on the interaction between magnitude and the side of presentation, creating an experimental procedure slightly different from SNARC. The idea here was to juxtapose the external presentation of a magnitude stimulus in terms of display space, to an assumed internal representation in mental space. In this sense, congruent and incongruent directional representations of the magnitude dimension could be created that would allow us to test hypotheses about spatial support of reasoning on order. In particular, we hoped from this methodology to be able to learn something about the above-mentioned asymmetry, that is, the cause for the one-sided “anchoring” of the dimensional poles themselves.

In setting up our experimental paradigm we aimed at staying fairly close to the original Huttenlocher (1968) and De Soto et al. (1965) methodology, in terms of not using pre-existing rank orders, such as the alphabet, the monthly sequence in the year, or numbers, as are commonly used in research on the SNARC effect. In such orders, that is, with pre-fixed sequences, the semantic asymmetry may well be triggered by extraneous factors, such as one might have read the monthly sequence “January, February, March,…” in writing, and so on. For us, the decisive question in this respect was whether even with materials that did not have a pre-fixed sequence *per se*, would we able to replicate the asymmetry, and what could be the underlying factor? This means, in other words, we used a methodology in which the analog representation of the rank order, be it spatially grounded or not, had no external counterpart, did not exist anywhere in the outside world, but was to be generated by the participants themselves, through integrative, transitive reasoning on a series of pairwise information.

In the first series of our own experiments (von Hecker et al., 2016), we had participants learn sets of ordered sequences of five persons, symbolized here as A–E. In a first learning phase, participants would view all ten possible pairs twice in a self-paced manner, in a random order. A pair always consisted of a dominant and a non-dominant person, with their positions on the screen (left or right during learning) counterbalanced. In order to clearly imply dominance, transitive comparators were used, e.g., “richer” or “taller,” such that “A > C” always held provided “A > B” and “B > C” was known. In an immediate test afterward, participants were presented with all possible pairs with the names appearing horizontally adjacent on the screen. In each test trial, participants saw the dominant element either appearing on the left, or on the right side of the display. The task was to indicate which of the two elements was the dominant one as quickly and as accurately as possible, using two marked, horizontally placed keys on the keyboard. First off, the SDE was replicated: Participants gave quicker, and more correct responses to queries on pairs of wider distances as compared to narrower distances, which we took as showing that an analog representation had been formed out of the initially learned piecemeal information. But furthermore, and consistent with Huttenlocher’s observation about an asymmetric tendency to mentally locate the array maximum to the left, we found a small, but reliable, *laterality effect*: A correct response was made more quickly when the dominant element in a pair appeared on the *left* side as opposed to the right side. We termed this tendency the Lateral Anchoring Effect (LAE). We concluded that the constructive process of integrating an array such as A – B – C – D – E out of the piecemeal information, participants tended to mentally locate, or *anchor*, the maximum (i.e., the most dominant element A) on the left side. In what way can such a laterality effect be a more valid indicator of spatial processes in linear order construction than the SDE?

A demonstration of lateral asymmetries can help bolster arguments in favor of an involvement of spatial processes in the construction of mental representations. As discussed above in relation to the SNARC effect for numerical stimuli (Gevers et al., 2005), such asymmetries may show up as compatibility between small and large magnitudes and left-right response key locations.

A second example for a spatial asymmetry underlying the mental, schematic simulation of an abstract order is time (Boroditsky and Ramscar, 2002). Participants in the West would see a temporal sequence of events (such as the meals of the day) as moving from left to right in time (Fuhrman and Boroditsky, 2010), which was reversed into right-to-left, in Hebrew-speaking participants. Notably, Tversky et al. (1991) found a strong tendency for children to arrange pictorial stories into sequences from left to right, yielding a temporal sequence of events. Whereas this tendency was clearly visible in English-speaking children, Arab and Hebrew children with a background in right-to-left-reading and writing, showed the opposite tendency. Preliterate kindergarteners, who had not been exposed to any fixed directional order of reading and writing, did not show any spatial biases (Dobel et al., 2007). This pattern of results gives a strong hint to the role of acquired skill in reading and writing, which seems to determine the direction in which the assumed spatial order representations unfold. We were thus led to assume that the reading-writing-direction (RWD) could causally determine the way in which our ordered mental models were constructed. Because reading and writing takes place, and is trained, as an activity in space, we assumed that the abstract template of this activity could be thought of, likewise, as a “template” for an abstract, dynamic concept. This could be an abstract template for how, in mental space, the constructive activity of putting piecemeal information together into an integrated, ordered array, may proceed.

Of course, the hypothetical qualification of RWD as a causal factor in mental model construction needed direct empirical support. In Experiment 7 of the above series (von Hecker et al., 2016) we replicated the described paradigm using an Iranian sample of participants with right-to-left RWD (Farsi). In this case, a “right-anchoring” effect is predicted, as corresponding to the “left-anchoring” we had observed in our Western participants. We compared a sample of University students with another sample drawn from a population that had not been exposed to Western RWD. In the student sample, the LAE was substantially weakened, that is, there was no trace of a right-anchoring, neither in terms of accuracy nor reaction times. On the other hand, the non-university sample did display our target “right-anchoring” effect. University students in Iran, we assumed, would have been exposed to Western RWD to some extend, using international literature and websites, this way assimilating left-to-right-RWD over time with practice. This might explain the pattern we found, as the clear LAE observed in the non-university participants could have been mitigated or completely washed out by the academic exposure, in the students. However in principle, as lateral anchoring was shown to reverse between left-to-right RWD backgrounds and right-to-left RWD backgrounds, we were now on more solid grounds in thinking about RWD as the triggering factor in the construction of spatial order representations.

As much as these results point to a cultural influence on the way spatial representations of order are formed, one should keep in mind that a fundamental connection between the abstract concepts of space and time (primacy) can be postulated on the basis of recent developmental findings. De Hevia et al. (2014) demonstrated that in a very early postnatal stage, 0- to 3-year old neonates showed reactions to a simultaneous increase (or decrease) in spatial extension and in duration (or numerical quantity). The neonates did however not react when the magnitudes varied in opposite directions^1^.

## Taking a Closer Look

Theoretically, the lateral anchoring phenomenon can inform a number of issues. First, the question arises that if it is the case that some spatial simulation underlies order construction in our participants, then what are the semantics of this simulation; in other words, what meaning does the constructed dimension have, what sort of metaphor does the constructed dimension engage that is being spatially simulated (see Lakoff and Johnson, 1980 for the role of metaphors in embodied cognition)? In this respect, one may consider two possibilities. Both assume different types of metaphors, but both have in common the assumption that people, when they construct a mental array, follow their learned RWD, that is for example, construction begins on the left side in English-speaking countries (see again Huttenlocher, 1968, p. 551).

*Possibility 1*: The spatial simulation of an ordered sequence such as *A is taller than B, B is taller than C, C is taller than D*, etc., may be based on *magnitude* as the underlying semantic dimension. Research from the number line and time line paradigms would both predict that if magnitude was correlated with quantification, people would preferably place the youngest (i.e., the “least tall”) on the left side and the tallest on the right side. In this way, higher numbers would represent larger quantity as being oriented toward the right, in the same way as allocating greater amounts of time passed toward the right side, as well.

*Possibility 2*: The spatial simulation of an ordered sequence such as *A is taller than B, B is taller than C, C is taller than D*, etc., may be based on primacy as semantic dimension, which itself may be based on dominance. Processing primacy (e.g., dealing with the first of a series of elements as derived from the action schema in RWD) may be seen as associated with dominance. Given this, one would predict that the tallest (i.e., the *most dominant* element) should be spatially positioned on the left side, with the least dominant element positioned on the right side. This converges with Casasanto’s (2009) argument that “Linguistic expressions like “the prime example” conflate primacy with goodness (i.e., this phrase can mean the first example, the best example, or both)” (p. 362). If primacy (as triggered by RWD) is in this way blended with dominance, thus yielding a “metaphorical blend” (Casasanto, 2009), one may expect the above.

These two possibilities make different assumptions about magnitude. The first possibility assumes that it is *magnitude* as such which is being mentally simulated. From this assumption it follows that the simulation would have to proceed from left to right (in the West) as the magnitude itself increases. The second possibility assumes that it is *primacy* which is mentally simulated. As part of this assumption, greater magnitudes should be represented on the left (as implying dominance) because there is a metaphoric blend between primacy and dominance. The simulation of lesser magnitudes would then proceed toward the right. We argue that the Lateral Anchoring Effect is in line with the second possibility. We submit that not magnitude *per se*, but *primacy* (as blended with dominance) is simulated via the spatial dimension. If primacy again follows the learned and trained RWD, then it would proceed from left to right in a Western population, and from right-to-left in the Iranian population. One earlier empirical finding clearly dovetails with these considerations. Previtali et al. (2010) studied the SNARC effect using newly acquired arbitrary sequences. This means that their randomly assembled series of stimuli (e.g., “bow,” “tent,” “apple,” “train,” etc.) did not have any implied order information apart from the explicit order information conveyed by the time sequence of their acquisition. The association that the authors found between the ordinal position of an item in the learning sequence and the respective spatial response preference in terms of the SNARC paradigm (see above) can be explained, in our terminology, as evidence for a spatial alignment of these items according to primacy: The earlier-encountered an item, the more to the left it was represented on the horizontal dimension of primacy.

Next, we were interested in situational influences on the constructive attempt in our participants. In other words, would the laterality effect be moderated by perceptual factors during learning? Therefore, in Experiment 2 (von Hecker et al., 2016), we introduced three conditions, whereby in the first, participants saw stimuli, during the learning phase, only with the dominant element on the left side (e.g., “A is *taller* than B,” Group 1). In the second condition, they saw stimuli only with the dominant element on the right side (e.g., “B is *less tall* than A,” Group 2). In the third condition, half and half of the stimuli were presented in either spatial orientation, randomly mixed (Group 3). We found that in Group 3, as in Group 1, participants exhibited a clear left-anchoring effect. That is, even in a situation (Group 3) where presentation conditions during learning could not cause any perceptual bias with respect to the spatial orientation of the to-be-constructed dimension, an LAE was observed. These results, and in particular the result from Group 3, are suggestive of a spontaneous tendency for anchoring. Later on, we replicated the LAE even in a situation where the names within a pair were presented centrally, one after the other, with the instruction that participants should understand the first-appearing name to be the dominant one. In addition, we also find the effect with central presentation when temporal ordering of the names is reversed (so that the second-appearing name is the dominant one) as well as when presentation order is randomized (von Hecker et al., 2019, see below).

Perceptual factors (e.g., a constant left-side presentation of the dominant element) are therefore unlikely to cause the laterality effect. Neither do we think, on the basis of these results, that the syntax structure as used in our previous experiments (= dominant element always on the left) caused the lateral bias. Instead, we submit that the construction of an analog representation of a rank order between a number of stimuli (Huttenlocher, 1968; Leth-Steensen and Marley, 2000; Sedek and von Hecker, 2004) is associated with a spatial process, as reflected in the consistently obtained left-anchoring phenomenon. RWD is, as we believe, the most likely trigger for this spatial process.

As another test of the robustness of lateral anchoring, we juxtaposed the directionality as implied by RWD, as presumably used by our participants to guide their spatial simulation, with a competing dimensionality, that is, the mental time line (see above, Dehaene et al., 1993; Fuhrman and Boroditsky, 2010). We know that the time line, in Westerners, is also oriented from left to right, but we made time-related information available that was orthogonal to dominance, so we could observe whether the RWD-guided constructive process was influenced by the time line (von Hecker et al., 2019). We used a centralized presentation, such that during learning, each name in a pair was presented, consecutively, at the center of the screen. The results were conclusive. First, when the dominant element was presented first in a pair during learning, thus being congruent with the presumed implication of the mental timeline, we observed a significant left-anchoring effect. Second, we also observed a significant left-anchoring effect in a condition in which the dominant element was randomly presented either first or second in a pair, thus making timeline-related information useless. Lastly, left-anchoring was also observed in a third condition, presenting the dominant element always second in a pair, this way consistently contradicting the implication from the mental timeline.

Our explanation so far makes one assumption that we thought was worth testing directly: We introduced “metaphorical blending” (see Casasanto, 2009) to mean that there occurs a blending between primacy and the meaning of the magnitude simulated on the particular dimension: Thus, to the extent that an element is see as the *taller*, *richer*, etc. in a pair, that same element attains the attribute of “dominant” and by blending, therefore, is seen as having more primacy. We see this blending as the cognitive basis of the observed lateral anchoring. To put it in simpler terms, RWD determines the direction into which array construction should proceed, whereas blending is supposed to determine which end of the magnitude dimension (i.e., minimum or maximum) is to be positioned at the origin of the constructed array.

It is precisely the “blending” assumption that we addressed in Experiment 4 of the series above (von Hecker et al., 2016). We tested the idea that dominance in a pair may derive from the semantics of the magnitude that the particular dimension represents. For example, if we assume that the meaning of *taller* can be described as “more on the height dimension” then the element in a pair that is described as “taller” will be the one that has “more height” and would therefore be seen as dominating the second element in the same pair which has “less height.” Note that *tall* is situated at the “unmarked” end of the height dimension, as compared to *short* which is situated at the marked end, see Hamilton and Deese, 1971). In this sense, “more” signifies an *asset of magnitude* on the height dimension. In a similar way, we can see all the remaining adjectives previously used in our studies, that is, *rich, old, smart, strong, fast*, as all reflecting an asset of magnitude on their respective dimension. This is different from comparing the elements within pairs with words such as *short, poor, young, dumb, weak*, and *slow*. These latter words, as their meanings highlight the marked end of the underlying dimension (see Hamilton and Deese, 1971), can be seen as representing a lack of magnitude on that dimension. The *shorter* person would not dominate the *less short* one, assuming that the relevant meaning of dominance relates to the matter of *magnitude*, and not to a purely grammatical meaning of dominance. And again, if we understand dominance as meaning an *asset* of a positive magnitude, we may predict that blending should be more easily possible between the meaning of primacy and the meaning of this particular positive magnitude, to blend into the semantics of primacy (as is the case in unmarked adjectives) as compared to a theoretically possible blending between primacy and any meaning that would refer to a *lack* in magnitude (as is the case in marked adjectives).

In our Experiment 4 we used unmarked and marked versions of the dimensions mentioned before, to compare in the light of the above predictions. Casasanto’s (2009) *metaphorical blend* assumption is in line with the prediction that metaphorical blending should predominantly happen when unmarked words are used. According to this, the lateral anchoring effect should be observable in this case. In this sense then, metaphoric blending could be seen as part of the explanation for this effect. It would also mean that one may see dominance as implying positive magnitude on the respective dimension, which means to understand dominance in terms of a simulated positive asset on the respective dimension (e.g., *richer*, *taller*, and *stronger*). On the other hand, metaphoric blending is less likely to occur between primacy and marked words (implying a lack of magnitude, e.g., *poorer*, *shorter*, and *weaker*). We therefore expected more of an observed lateral anchoring effect in the condition that used unmarked, as compared to marked comparator words. With marked comparators, on the other hand, only a weak, or even non-existing left-anchoring should be observed. The results were clear. There was a replication of the left-anchoring effect in the group where we used unmarked comparators, thus implying a meaning of asset. However in the group where we used marked comparators, thus implying a lack of asset, there was no trace of the laterality effect. This gave reason to assume that a lack of asset, or magnitude, on one side, and primacy on the underlying dimension on the other side, are both not compatible for blending since they are not congruent in their meaning (cf. Casasanto, 2009). Research has shown, however, that tasks that require a judgment at the marked end of a dimension tend to be more difficult than tasks that direct participants’ processing to the unmarked end (Sherman, 1973, 1976; Hines, 1990). This could have influenced the above results because when using marked comparators, error variance might have obscured a possibly existing left-anchoring effect. However, the fact that in the group using marked comparators, latencies for responses were found to be not longer than in the group using unmarked comparators, counts against this possibility. In both groups, the SDE in terms of correct responses and reaction times was replicated.

To summarize the interpretations from our LAE paradigm so far, we submit that lateral anchoring may constitute an empirical signature effect to support our general argument about the construction of a spatially extended, analog representation of order (left-to-right in Westerners, right-to-left in Iranians). When studying a sequence of pairwise relationships, expressing a transitive rank order between five elements, A.E, a participant will attempt to build a mental array, thus forming a spatial representation (see De Soto et al., 1965; Huttenlocher, 1968; Potts, 1972, 1974; Leth-Steensen and Marley, 2000). In doing so, the left side or the right side, depending on the persons trained RWD, is taken as the side of origin for the to-be-constructed array. By means of applying transitivity and shifting of elements (see above) one would then position the maximum element of the rank order at that origin (spatially *left* in Westerners, and *right* in Iranians), which would serve as anchoring of the dimension. Model construction then unfolds in *rightward*/*leftward* direction, depending on RWD. In a later test on any particular pair in the rank order, one might then recollect and activate the so-constructed spatial representation. When the spatial arrangement in the queried pair (on the screen) shows the dominant element (person) on the side that is close to the perceiver’s dimensional origin, both representations – the one on the screen and the perceiver’s mental model – are spatially congruent, and a correct response may be quickly made. However, if the side of presentation of the dominant element is opposite to the perceiver’s dimensional origin, interference created by this incongruity will slow correct responses down, or even affect the accuracy of the response.

## Relation to Mental Model Theory

The above interpretations lead naturally to a discussion of the Model Theory of Relational Reasoning (see, e.g., Johnson-Laird and Byrne, 1991). We center this discussion around the five assumptions put forward by Goodwin and Johnson-Laird (2005) to explain reasoning with relations. For each of these five assumptions, we discuss how the interpretation of the LAE and the pertaining results relate to the particular assumption.

Assumption 1, Iconicity: “Models are iconic in that their parts and relations correspond to those of the situations they represent.” (p. 467). We submit that the character of iconicity in a constructed model of a hierarchical rank order is less one of mental imagery, possibly containing particular points of view, perspectives, or metrics (Kosslyn, 1980; Finke and Shepard, 1986), but is more one of an abstract representation, wherein spatial relations between the entities are preserved in the model, that is, on an analog dimension.

Assumption 2, Emergent consequences: “Individuals use the meanings of relational assertions in intensional representations to construct mental models of the extensions of assertions, and the logical consequences of relations emerge from these models.” (p. 467)

According to our own assumptions, the meaning of an order relation (in Goodwin and Johnson-Laird’s terminology, its “intension”) is being abstracted in terms of primacy. That is, a reasoning individual will use the meaning of a comparator such as “rich” by simulating a continuum of richness, within which “the richest” element in the order is assigned highest primacy. The intensional representation is important for the construction of the order model because it conveys the anchoring point. That is, the order will be constructed starting at the origin defined by “the richest,” and onward in the direction of less rich elements within the order. In this process the intensional meaning is particularly important because it not only engenders the attribution of primacy to “richer” elements compared with “less rich” elements as just explained, but also warrants the blending with the abstract notion of “procedural primacy” as derived from the trained RWD action model, so that the origin may be placed, for example, on the left side in Westerners.

Assumption 3, Parsimony: “Individuals tend to construct only a single mental model of a set of relations, to construct the simplest possible model, and to use their knowledge to yield a typical model.” At present, this assumption dovetails with the assumptions made for the LAE to occur insofar as we believe that one single model representing an order hierarchy is constructed and integrated on the basis of pairwise, transitive pieces of information (e.g., A > B, B > C, C > D, and so on). The findings on the LAE are, yet, silent in respect to Assumption 3 when it comes to more complicated situations, such as more complex semi-orders, or situations in which the number of to-be-learned elements in the order would introduce issues of resource limitation in working memory (see Klauer and Stegmaier, 1997). Such situations await investigation.

Assumption 4, Strategic assembly: “Naive reasoners assemble reasoning strategies from an exploration of different sequences of tactical steps.” (p. 479) Strategies for anchoring still await empirical investigation. At the most general level, we predict that such strategies would theoretically comprise all heuristics that would help to derive primacy, as primacy stands at the core of establishing a meaningful order representation via blending (see Assumption 2). For example, if A > B is presented first, and C > D second, then the relation between B and C is indeterminate at that point in the process of learning. If no further information on this relation is given, a learner, in order to construct a complete, integrated spatial model, may use the temporal sequence of presentation as proxy for space. They might therefore construct the “chain” A – B – C – D by mapping the time line onto the spatial dimension under construction, despite the fact that the logically necessary “adjacent” information B > C had not at all been presented.

Assumption 5, Integration: “The more complex the integration, the harder the task should be. This complexity depends on the number of entities that need to be integrated […] and on the depth of the relation over these entities.” (p. 480). Integration is assumed to be possible by way of applying transitivity to the elementary pairwise relationships. That is, if it is known that A > B, and that B > C, then integration may take place in terms of using the common term “B” to merge both pieces of information into an analog representation isomorphic to a “chain,” A-B-C. Two consequences arise from this. First, the task will be harder the more elementary pairwise relationships have to be integrated in this way. Second, assuming a given number of elements in the hierarchical order, the task should become easier (as compared to a situation in which only “adjacent” elements are presented for learning) the more redundant, transitive information is given in addition. For example, integrating a “chain” like A – B – C – D – E should be easier if at least some transitive information, e.g., A > C and C > E, is already provided during learning, as compared to a learning situation in which only adjacent information, e.g., A > B, B > C etc. is presented. Assuming further that the attribution of primacy and the process of blending (see Assumption 2) both depend on integration as just described, we predict that the emergence of an LAE will also be moderated by how successful the piecemeal information was integrated into the overall representation.

## Conclusion

The ability to infer transitivity from pairwise ordered relations is a fundamental cognitive faculty which can even be observed in non-human species (e.g., Delius and Siemann, 1998; Lazareva et al., 2004). In the present research we are interested in exploring the characteristics of mental representation when such transitive relations are learned in humans. For order hierarchies, including temporal/serial orders in listings or in sequences of acquisition, recent research supports the idea that people tend to keep such information in an activated state in working memory by means of spatial representations (e.g., Abrahamse et al., 2014). In verbal list learning, people from Western backgrounds tend to associate the items at the beginning of a list with the left side, and those at the end with the right side (e.g., van Dijck and Fias, 2011; Guida et al., 2016). Notably, this basic phenomenon also extends to sensory-deprived populations, such that spatial processing in the context of establishing order representations does not seem to critically depend on sensory experiences (Rinaldi et al., 2018).

The LAE as presented here links up with a wide range of literature converging on the idea that spatial representations of numerical and non-numerically ordered information underlie transitive reasoning (Hubbard et al., 2005; Fias et al., 2007; Zorzi et al., 2011; Mathieu et al., 2015; Alfred et al., 2020, and many more). Much of the recent evidence comes from neuroimaging studies which, as discussed above, have recently benefited from a number of methodological refinements, thereby sharpening the argument about numerical and otherwise ordered information being processed in areas within the parietal lobe. As such, the LAE contributes a behavioral paradigm complementing the neurophysiological argument by providing data referring to a fundamental behavioral indicator of spatial processing and spatial representation: the left-vs-right dimension in subjective space (Klatzky, 1998; Burgess, 2006).

Theoretically, we believe that the SNARC effect and the LAE both rely on mental model based mechanisms. In this respect, the SNARC effect reveals an interplay between a mental representation of the “number line” on the one hand, and a lateralized motor response on the other hand, whereas the LAE reveals an interplay between an *ad hoc*-constructed mental model of an ordinal hierarchy on the one hand, and a directional arrangement of elements within a shown test pair, as such representing a particular direction (from maximum to minimum), that is, the overall directionality of the whole hierarchy, in terms of display.

In presenting data which we interpret as supporting spatial processes in rank order reasoning we aimed at not re-instating the Homunculus flavor into our explanation of the SDE^2^. Rather, our aim was to build a bridge between the classical, self-report-based, observations by Huttenlocher (1968) and a new experimental paradigm that may, with some justification, be interpreted as independently supporting the involvement of spatial processes in rank order reasoning. We wanted to avoid the fallacies of self-report as a methodology, such the possibility of incomplete recollection or reconstructive strategies being used at the time of giving the report, possibly coloring the description of any psychological processes that presumably took place at the time of an actual trial. In the end, people are not necessarily good reporters on the mechanics within their own cognitive system (Nisbett and Wilson, 1977). We also wanted to avoid the fallacy of reverse inference with respect of the available neurophysiological data that had given rise to spatial interpretations of the SDE in the past. But obviously, in presenting an interpretation as we did, that is, in pointing to – what we call – spatial involvement in rank order reasoning in terms of the LAE, the question arises what we actually mean by “spatial involvement.” Are we now closer to an independent experimental validation of Huttenlocher’s self-report observations than before? The answer to this question, as we think, comes in the shape of one conceptual remark, and one new question.

First, as a conceptual remark, one may prefer to revisit the scientific meaning, and use, of the term “spatial process.” The term as such implies some reference to colloquial understanding about what the experience of “space” means, as such experience is fundamental to human perception and behavior (Kant, 1999). As such, the term serves to label a type of process about which, at least in terms of its neural correlate, we know relatively little. On the other hand, we know from empirical studies that there are important unconscious aspects of mental activity (see Nisbett and Wilson, 1977), as different, and contradictory, spatial information can be part of memory contents in cognitive and, simultaneously, sensori-motor visual systems (e.g., Bridgeman, 1992). Therefore, we do not claim conscious experience to be a necessary part of our understanding of “spatial process.” Rather, it may be, and is highly likely, that certain brain regions that have been associated with spatial processing are also centrally involved in other, colloquially different, brain activity. For example, results from neurophysiology suggest that the Inferior Parietal Lobule (IPL) is involved in mechanisms decision making. Activation in the IPL is involved in spatial behavior. In monkey models task-related activation patterns after spatial stimulation were found even for single neurons in the IPL area (Andersen et al., 1985). The IPL has also be found to be involved in decision making under uncertainty, using in different input modalities, auditory and visual (Vickery and Jiang, 2009). This seems to imply that regions such as the IPL, although centrally involved in “spatial processes” as we may describe them colloquially, may serve a much more generic function in the context of a larger functional network. This generic function however might be insufficiently described (in terms of its to-be-assumed generality) when giving it the otherwise plausible, colloquial label of “spatial.”

The second part of the answer asked above comes as a new question. The question is, can we find a neurophysiological correlate to the lateral anchoring effect? In order to further specify the functional networks in the brain which serve reasoning on hierarchical magnitudes and ranked orders, can we substantiate the claim that the representations we are investigating share some neural substrate with other tasks which, at the colloquial level, would be given the label “spatial”? Reverse inference does not have to be invoked in a situation where, as is true in case of the LAE, the label “spatial” can already be assigned to the target task *a priori*, so does not need to be inferred. The theoretical link between the LAE and the trained RWD, as we argue, licenses such an assignment *a priori*. Research in this direction is currently on its way.

## Author Contributions

UvH provided a first draft and finalized the manuscript. KCK commented on that draft. Both authors contributed to the article and approved the submitted version.

## Conflict of Interest

The authors declare that the research was conducted in the absence of any commercial or financial relationships that could be construed as a potential conflict of interest.

## Footnotes

1 In an extension of this research, de Hevia et al. (2017) found that at birth humans associate numerical order with different spatial locations (smaller on the left and larger on the right). Different from the mainstream of results reviewed here, though, these effects pertain to concrete magnitudes, involving numerosity. The exact mechanisms that bring them about still have to await further exploration. There are hints, mainly from research on non-human species, pointing toward hemispheric dominance effects playing a role.

2 It should be noted that within another, related class of paradigms, i.e., equivalence learning, distance effects in reversal of the SDE are sometimes found (Bentall et al., 1999; Dickins, 2005). In these studies, participants are first similarly trained on a series of AB, BC, CD, and DE pairs, etc., until all pairings are thoroughly learned. Typically then in a test phase, accuracy and response speed for correct responses decrease with pair distance within the chain. In comparing the two paradigms, one possibility to explain these differences could be the way the learning phase is structured in both cases: Whereas in our studies, all possible pairwise relations are learnt (“adjacent” pairs as well as wider pairs), in the equivalence learning trials only adjacent pairs of the chain are presented for learning. This means that the wider pair relations, as implicated by transitivity, have to be construed at test from the original piecemeal information, which is costly in terms of response speed and accuracy. The fact that in this paradigm, the “inverse SDE” diminishes with increasing practice (Spencer and Chase, 1996; Bentall et al., 1999) might strengthen this explanation. Other possible explanations for a reversal of the SDE, pertaining to the nature of the spatial representation in order relations versus equivalence relations, await further research. In particular, further research might contrast the different learning regimes employed here and in the above-mentioned work both for the learning of equivalence relations leading to a reversed SDE and the learning of dominance relations leading to a regular SDE effect.
